# Medicaid Coverage Policy Variations for Chronic Pain and Opioid Use Disorder Treatment

**DOI:** 10.1001/jamanetworkopen.2025.26796

**Published:** 2025-08-13

**Authors:** Meredith C. B. Adams, Seth M. Eller, Cara McDonnell, Sarjona Sritharan, Rishika Chikoti, Amaar Alwani, Elaine L. Hill, Robert W. Hurley

**Affiliations:** 1 Department of Anesthesiology, Wake Forest University School of Medicine, Winston-Salem, North Carolina; 2 Center for Artificial Intelligence, Wake Forest University School of Medicine, Winston-Salem, North Carolina; 3 Department of Public Health, Wake Forest University School of Medicine, Winston-Salem, North Carolina; 4 Pain Outcomes Laboratory, Wake Forest University Health Sciences, Winston-Salem, North Carolina; 5 Department of Translational Neuroscience, Wake Forest University School of Medicine, Winston-Salem, North Carolina; 6 Department of Public Health Sciences, University of Rochester Medical Center, Rochester, New York

## Abstract

**Importance:**

Co-occurring chronic pain and opioid use disorder (OUD) are associated with a high disease burden for the patient, requiring comprehensive treatment approaches, yet Medicaid benefit structures for evidence-based therapies vary substantially across states.

**Objective:**

To develop a systematic framework for analyzing Medicaid coverage policy variations in behavioral and integrative health services for co-occurring chronic pain and OUD.

**Design, Setting, and Participants:**

This systematic economic evaluation reviewed Medicaid coverage policies in California, Illinois, Texas, North Carolina, and New York from January 1, 2018, to December 31, 2023, representing states with a combined Medicaid enrollment of approximately 27.8 million beneficiaries in 2018 and growing to 35.9 million by 2023. Findings were applied to demonstrate methodology for future comparative policy effectiveness research.

**Exposures:**

Medicaid coverage and co-occurring chronic pain and OUD.

**Main Outcomes and Measures:**

Coverage status, authorization requirements, service limitations, and clinician qualifications for medications, behavioral health services, and integrative treatments.

**Results:**

Five states covering approximately 35.9 million Medicaid beneficiaries at peak enrollment were analyzed. All states provided full coverage for core medication classes and basic interventional procedures, although with varying authorization requirements. Behavioral health coverage showed policy divergence: peer support services were universally covered but differently implemented, with Texas limiting coverage to 104 units per 6 months. Four states (80%) covered cognitive behavioral therapy with varying session limits. Physical therapy was universally covered, while only 2 states (40%) provided acupuncture coverage.

**Conclusions and Relevance:**

In this economic evaluation, substantial Medicaid implementation variations were documented across behavioral and integrative health services, establishing foundation methodology for future comparative effectiveness research to examine relationships between policy approaches and patient outcomes.

## Key Points

**Question:**

How do Medicaid coverage policies for behavioral and integrative health services vary across states for treating co-occurring chronic pain and opioid use disorder?

**Findings:**

In this economic evaluation of 5 populous US states from 2018 to 2023, universal coverage existed for core medications and basic interventional procedures, while substantial implementation variation occurred in behavioral health services and integrative therapies, with acupuncture coverage limited to 2 states.

**Meaning:**

These findings suggest that variation in Medicaid policy implementation creates a foundation for understanding potential access disparities, requiring future research to evaluate patient outcomes.

## Teaser

This economic evaluation systematically examines Medicaid policies governing behavioral and integrative health services for treating opioid use disorder and chronic pain across 5 populous US states.

## Introduction

The co-occurrence of chronic pain and opioid use disorder (OUD) presents significant clinical challenges, with each condition potentially amplifying the severity and complicating the treatment of the other. Patients with co-occurring chronic pain and OUD experience higher disease burden and require more intensive health care services compared with those with either condition alone.^1^

Behavioral and integrative health models have emerged as promising strategies for treating co-occurring chronic pain and OUD. These approaches combine conventional interventions with psychotherapy and complementary treatments such as acupuncture to address both conditions simultaneously. The effectiveness of integrated approaches is particularly relevant given high psychiatric comorbidity rates in this population,^2^ making psychotherapeutic interventions and peer support services essential components.^3^

The National Institutes of Health’s IMPOWR (Integrative Management of Chronic Pain and OUD for Whole Recovery) research network is evaluating integrated behavioral and medical approaches for these concurrent conditions.^4^ However, insurance coverage limitations often impede adoption of evidence-based interventions in routine clinical care.

Medicaid, as the largest payer for substance use disorder (SUD) treatment and mental health services in the US, plays a crucial role in determining treatment access.^5^ Recent policy reforms have expanded coverage for residential treatment and medications for OUD while reducing preauthorization requirements.^6^ However, significant variation exists across state Medicaid programs in coverage for behavioral health services and nonpharmacologic pain treatments,^7^ potentially creating disparities in access.

This economic evaluation systematically examines Medicaid policies governing behavioral and integrative health services for treating OUD and chronic pain across 5 populous states: California, Illinois, Texas, North Carolina, and New York. By analyzing coverage variations, we aim to establish a foundation for understanding policy approaches and identify areas requiring future outcomes research.

## Methods

### Ethical Considerations

This economic evaluation involved only publicly available policy documents and did not require institutional review board approval per Wake Forest University policy for studies using exclusively public documents without identifiable information. No informed consent was required as no human participants were involved. This report follows applicable components of the Consolidated Health Economic Evaluation Reporting Standards (CHEERS) reporting guideline for health economic evaluations, specifically systematic policy review methodology elements.

### Study Scope

This analysis examines policy structures and coverage specifications but does not evaluate patient outcomes, access patterns, or clinical effectiveness. The study provides a descriptive framework for understanding policy variation rather than assessing the impact of different approaches on patient care delivery or health outcomes.

### Data Collection

We selected 5 study states to achieve widespread geographic coverage and represent diverse policy approaches and regional variations: California,^8^ Illinois,^9^ North Carolina,^10^ New York,^11^ and Texas.^12^ We conducted systematic reviews of each state’s Medicaid website to identify coverage for peer support services, nonsteroidal anti-inflammatory drugs (NSAIDs), corticosteroids, anticonvulsants, antidepressants, cognitive behavioral therapy, physical therapy, chiropractic services, opioids, interventional therapies, and acupuncture.

Coverage was assessed from January 1, 2018, to December 31, 2023, using historical data through website archives and clinician manuals. Categories included (1) not covered, (2) covered by fee-for-service, (3) covered by managed care organization, (4) covered but unspecified, and (5) not found.

### State-Specific Methods 

North Carolina implemented a Fee Schedule & Covered Code Portal^13^ in November 2022, requiring use of fee schedule archives^14^ for earlier periods. Texas^15,16^ and Illinois^17,18,19^ had comprehensive fee schedules, while New York^20^ and California^21^ required additional external resources including commercial websites to assess clinic-level Medicaid acceptance. Preferred drug lists were used to identify medication coverage and stipulations,^22,23,24,25,26^ with longitudinal data accessed through archived preferred drug lists when available.

### Statistical Analysis

This study employed descriptive analysis of policy coverage patterns across states and treatment categories. Coverage patterns were summarized using frequencies and percentages. No inferential testing was performed, as this was a qualitative policy analysis examining regulatory frameworks rather than patient outcomes..

## Results

This systematic policy analysis examined Medicaid coverage policies across 5 states serving approximately 35.9 million beneficiaries at peak 2023 enrollment (California, 16.0 million^27^; New York, 8.1 million^28^; Texas, >5.0 million^29^; Illinois, 4.0 million^30^; North Carolina, 2.9 million^31^). The study states represented diverse geographic regions and policy approaches.

### Coverage Consistency for Core Services

All 5 states (100%) demonstrated full coverage for fundamental medication classes essential to pain management (Table 1). This included complete coverage of NSAIDs, with consistent inclusion of common agents such as ibuprofen, naproxen, and diclofenac across all states. Similarly, universal coverage extended to corticosteroids (both oral and injectable formulations), anticonvulsants (particularly gabapentin and pregabalin), and antidepressants (including both selective serotonin reuptake inhibitors and serotonin-norepinephrine reuptake inhibitors). While all states covered both short- and long-acting opioids, they implemented different prior authorization requirements and quantity limitations as detailed in Table 1.

**Table 1.** Core Service Coverage Patterns and Implementation Requirements Across State Medicaid Programs, 2018 to 2023^a^

Service category	State				
	Illinois	North Carolina	Texas	California	New York
**Pain medications**					
NSAIDs	Covered; 23 preferred agents	Covered; 9 preferred agents	Covered with quantity limits	Covered with step therapy	Covered with PA for select agents
Opioids	Covered with PA	Covered with quantity limits and PA	Covered with strict PA	Covered with medical necessity	Covered with strict quantity limits
**Basic interventional procedures**					
Epidural injections	Covered; PA for repeat	Covered; PA for multiple levels	Covered with medical necessity	Limited to 3 to 6 mos	Covered with PA
Facet (spine osteoarthritis) procedures	Covered with documentation	Maximum 2 levels/session	Covered with PA for therapeutic (RF)	Limited frequency	Covered with PA
**Behavioral health**					
Peer support	SUD treatment only	Comprehensive services	104 units/6 mos	Covered under HCPCS code H0038	Covered under HCPCS code H0038
CBT	Individual/group covered	Part of SUD services	30 visits/y	Under *CPT* 98978	30-, 45-, or 60-min sessions

Abbreviations: CBT, cognitive behavioral therapy; *CPT*, *Current Procedural Terminology*; HCPCS, Healthcare Common Procedure Coding System; NSAIDs, nonsteroidal anti-inflammatory drugs; PA, prior authorization; RF, Radiofrequency ablation; SUD, substance use disorder.

^a^ Preferred agents refer to medications covered without prior authorization. PA indicates required preapproval for service delivery. Medical necessity requirements indicate the need for documented clinical justification. HCPCS code H0038 refers to “self-help/peer services, per 15 minutes.” *CPT* code 98978 refers to “remote therapeutic monitoring (eg, respiratory system status, musculoskeletal system status, therapy adherence, therapy response); device(s) supply with scheduled (eg, daily) recording(s) and/or programmed alert(s) transmission to monitor cognitive behavioral therapy.”

Table 1 demonstrates that while coverage existed universally, the states implemented markedly different gatekeeping mechanisms. Illinois maintained the most permissive approach with 23 preferred NSAID agents available without authorization, while New York required prior approval for select agents. Similarly, opioid coverage revealed each state’s distinct philosophy toward controlled substance management. Texas imposed the strictest prior authorization requirements, whereas North Carolina focused primarily on quantity limitations with prior authorization protocols.

Basic interventional procedures showed similar consistency in coverage. All states (100%) provided coverage for highly used interventional pain procedures, including epidural corticosteroid injections (cervical, thoracic, and lumbar), facet joint injections, medial branch blocks, peripheral nerve blocks, major joint injections, and trigger point injections (Table 1). Documentation requirements varied significantly, with Texas requiring specific medical necessity documentation and North Carolina requiring prior authorization for multiple-level procedures.

Authorization requirements and service limitations showed substantial variation across programs. Prior authorization was commonly required for multiple-level procedures, repeat procedures, and advanced interventional techniques. Medical necessity documentation requirements were universal but varied in specificity and scope. Time-limited authorizations and quantity limits were prevalent, particularly for behavioral health services and certain medication classes.

### Behavioral Health Integration

The framework application revealed systematic patterns in behavioral health service coverage implementation, with universal peer support coverage masking substantial structural differences in service delivery specifications (Table 2). All 5 states provided coverage for peer support services, although with distinct specifications. Illinois limited coverage to SUD treatment support, while North Carolina offered more comprehensive peer services, including recovery coaching, skill-building groups, and community resource connections for SUD. Texas implemented specific unit limitations of 104 units within 6 months. In the context of Medicaid billing and service limitations, a unit typically refers to a standardized increment of time or service. Peer support services are often billed in 15-minute increments, corresponding to Healthcare Common Procedure Coding System code H0038 (“self-help/peer services, per 15 minutes”). Therefore, 104 units would equate to 26 hours of service.

**Table 2.** Behavioral Health Service Coverage Specifications by State^a^

State	Peer support services	Service limitations	CBT coverage	CBT limitations
Illinois	SUD treatment support only	Treatment setting restricted	Individual and/or group skill building	Must address psychiatric disabilities
North Carolina	Full coverage including coaching	Must be in treatment plan	Part of outpatient SUD	Requires ASAM assessment
Texas	Mental health and SUD	104 units/6-mo period; maximum 12 in group	Individual/group therapy	30 visits/y, 4 h/d
California	Under HCPCS codes H0038 and H2014	Licensed clinician supervision	Under *CPT* code 98978	Physician prescription required
New York	Self-help and/or peer services	Must be in treatment program	30-, 45-, and 60-min sessions	Maximum 8 persons per group

Abbreviations: ASAM, American Society of Addiction Medicine; CBT, cognitive behavioral therapy; *CPT*, *Current Procedural Terminology*; HCPCS, Healthcare Common Procedure Coding System; SUD, substance use disorder.

^a^ Treatment setting restrictions indicate services must be delivered in specific clinical environments. ASAM assessment refers to criteria-based evaluation required for service authorization. Licensed clinical supervision requirements specify oversight by qualified mental health professionals. HCPCS code H0038 refers to “self-help/peer services, per 15 minutes” and H2014 indicates “skills training and development, per 15 minutes.” *CPT* code 98978 refers to “remote therapeutic monitoring (eg, respiratory system status, musculoskeletal system status, therapy adherence, therapy response); device(s) supply with scheduled (eg, daily) recording(s) and/or programmed alert(s) transmission to monitor cognitive behavioral therapy.”

Cognitive behavioral therapy (CBT) services were covered by 4 of the 5 states (80%), with implementation heterogeneity as detailed in Table 2. Illinois covered individual and group skill-building and cognitive behavioral interventions, while North Carolina included CBT within outpatient SUD services, suggesting it is primarily intended for OUD treatment. Texas imposed a limit of 30 visits per calendar year without explicitly stating the indication, and New York specified coverage for 30-, 45-, and 60-minute sessions. California provided coverage under specific *Common Procedural Terminology* (*CPT*) codes.

Table 2 reveals how states operationalized behavioral health integration differently despite universal peer support coverage. Texas’s restriction to 104 units per 6 months (equivalent to 26 hours of service) represented the most conservative approach, while North Carolina offered the most comprehensive model with recovery coaching and community resource connections. The CBT landscape showed even greater divergence. Illinois covered both individual and group interventions without explicit visit limits, while Texas capped coverage at 30 visits annually, suggesting fundamentally different assumptions about treatment duration and intensity needs.

### Integrative Care Coverage Patterns

Integration of complementary therapies (eg, physical therapy, acupuncture, and chiropractic services) showed the greatest variation among covered services (Table 3). Physical therapy services were universally covered (100%), although with different limitations. Illinois and New York implemented no explicit visit limits, while Texas required specific documentation of medical necessity. Chiropractic services were covered by 4 states (80%), with coverage specifications ranging from 12 visits in 12 months in Texas to coverage based on specific *CPT* codes in California (Table 3).

**Table 3.** Integrative Therapy Coverage and Restrictions by State^a^

State	Physical therapy	Chiropractic services	Acupuncture	Documentation requirements
Illinois	Full coverage	Coverage in 2021 only	Coverage in 2023 only	Must show functional improvement
North Carolina	All standard modalities	Requires x-ray documentation	Not covered	Medical necessity documentation
Texas	Complete coverage	Limited to 12 visits/12 mo	Not covered	Limited to acute conditions
California	Based on medical necessity	Covered under specific *CPT* codes	Full coverage	Requires physician prescription
New York	No explicit visit limits	Not covered	Not covered	Must demonstrate progress

Abbreviation: *CPT*, *Current Procedural Terminology*.

^a^ Functional improvement documentation requires evidence of progress in specific physical or functional metrics. Medical necessity documentation indicates the need for clinical justification of ongoing treatment. Acute condition limitations restrict coverage to short-term treatment of new or recently exacerbated conditions. Physician prescription requirements indicate services must be ordered by a licensed physician. *CPT* codes for chiropractic services in California include specific spinal manipulation codes (98940-98942) and therapeutic procedure codes (97110-97140).

Acupuncture demonstrated the most limited coverage, with only 2 states (40%) providing coverage. Illinois restricted coverage to 2023 only, while California covered services under specific *CPT* codes as detailed in Table 3. Other integrative services showed variable coverage patterns, with osteopathic manipulation covered by 4 states (80%) but excluded by North Carolina. Exercise therapy generally received limited coverage and was often bundled within physical therapy services.

Table 3 exposes the most pronounced coverage disparities in our analysis. While physical therapy achieved universal coverage, the implementation approaches ranged from Illinois and New York’s unlimited access to Texas’s documentation-intensive model requiring specific medical necessity justification. The acupuncture findings are particularly striking: only Illinois and California provided coverage, with Illinois limiting access to 2023 only, potentially reflecting pilot program experimentation. This pattern suggests that states viewed integrative therapies as optional enhancements rather than core treatment components, despite growing evidence supporting their effectiveness in pain treatment.^32^

### Authorization and Documentation Requirements

A consistent pattern emerged regarding authorization requirements across service categories (Tables 1-3). Prior authorization was commonly required for long-acting opioids, multiple injection procedures, extended therapy courses, and nonpreferred medications. Documentation requirements varied but typically included a demonstration of medical necessity, functional improvement, failed conservative treatment, pain scores and functional assessments, and treatment plan updates.

These policy variation patterns established baseline documentation necessary for future research examining relationships between coverage approaches and patient outcomes, while highlighting areas where implementation differences may warrant empirical evaluation. The variation in implementation strategies and coverage specifications suggested opportunities for policy standardization while highlighting the challenges of balancing access with appropriate use management.

## Discussion

Our systematic economic evaluation reveals both promising developments and persistent challenges in Medicaid coverage for treatment of co-occurring OUD and chronic pain. Universal coverage of fundamental medications and basic interventional procedures provides a foundation for care, but varying implementation approaches create a complex landscape requiring further investigation.

### Policy Variation Framework

The documented differences in behavioral health service implementation create natural experiments for evaluating relationships between policy structure and care delivery outcomes. These variations enable future research examining whether different peer support service specifications or CBT coverage approaches affect treatment engagement or clinical outcomes. As Grogan et al^7^ previously identified, such disparities in Medicaid coverage for substance use treatment can significantly impact treatment outcomes. Our findings align with those of Busch et al,^33^ who noted limited success in early efforts to enhance mental health management in integrated care settings. The varying coverage patterns for behavioral health services, particularly the distinct specifications for peer support services and CBT, suggest challenges in standardizing integrated care delivery. Different clinician credential requirements, supervision specifications, and setting restrictions may complicate coordinated care delivery, although the impact on patient outcomes requires empirical evaluation.

### Coverage Implementation Heterogeneity

The varying coverage patterns for behavioral health services, particularly the distinct specifications for peer support services and CBT, demonstrate challenges in standardizing integrated care delivery approaches. These implementation differences reflect varying state priorities and resource allocation strategies rather than necessarily indicating access barriers.^7^

### Areas for Future Investigation

Documentation of limited acupuncture coverage in 2 of 5 states and varying chiropractic care specifications provides a foundation for future research examining whether expanded integrative therapy coverage affects pain outcomes or health care utilization patterns among patients with co-occurring conditions. The evidence base for these interventions in treating co-occurring chronic pain and OUD^1^ suggests opportunities for investigating whether expanded coverage translates to improved clinical outcomes.

### Implications

This systematic economic evaluation documents substantial variation in Medicaid implementation approaches for behavioral and integrative health services treating co-occurring chronic pain and OUD. While coverage for fundamental treatments demonstrates consistency across states, implementation heterogeneity in behavioral health services and integrative therapies creates a complex policy landscape requiring empirical investigation. The methodological framework established herein provides a foundation for future comparative effectiveness research examining how documented policy differences affect patient access, care coordination, and clinical outcomes. Such research will be essential for evidence-based policy development and standardization efforts.

The policy variation documented herein extends beyond administrative differences to represent fundamentally different philosophies of care delivery. States with broader coverage demonstrate recognition that co-occurring conditions require integrated treatment approaches, while more restrictive policies may reflect resource constraints or skepticism about nontraditional interventions. This divergence has immediate implications for the 35.9 million Medicaid beneficiaries in our study states and millions more nationwide.

Most critically, our findings suggest that current policy variations may perpetuate health disparities based on geography rather than clinical need. A patient’s access to evidence-based integrated care should not depend on their state of residence. The documentation of these disparities establishes an urgent research agenda: determining which policy approaches optimize patient outcomes while maintaining fiscal responsibility.

As states continue refining their Medicaid policies, the framework established herein provides a roadmap for evidence-based decision-making. The documented policy variations suggest several areas for future empirical investigation: whether standardized authorization approaches improve care coordination while maintaining appropriate oversight, whether expanded coverage for evidence-based integrative therapies demonstrates cost-effectiveness in this population, and whether different clinician qualification requirements affect care quality and network adequacy. These issues require controlled evaluation before policy standardization recommendations can be supported. The ultimate goal must be ensuring that all patients with co-occurring chronic pain and OUD have access to the comprehensive, integrated care that evidence shows they need to achieve sustainable recovery and improved quality of life.

The opportunity for policy leadership in this area is significant. States that invest in comprehensive, integrated coverage models today may not only improve patient outcomes but also establish sustainable care delivery systems that other states can adopt. This analysis provides the foundation for that evidence-based policy evolution.

### Areas for Future Policy Research

The policy variations documented in this analysis suggest several priorities for future empirical research. Comparative effectiveness studies should examine whether different authorization approaches affect care coordination and patient outcomes while maintaining appropriate oversight. Longitudinal research investigating the clinical effectiveness and cost-effectiveness of expanded coverage for evidence-based integrative therapies could inform policy decisions about coverage expansion. Studies comparing different clinician qualification requirements could identify approaches that optimize care quality while maintaining adequate network capacity.

### Limitations

This study has limitations. The data represent coverage policies but do not address reimbursement rates, which create additional access considerations for patients with Medicaid coverage.^34,35^ This study describes policy variation but does not evaluate patient outcomes or access impacts, which require future empirical research. This analysis focused on policy structures rather than implementation effectiveness, utilization patterns, or clinical outcomes.

Analysis was limited to 5 states, potentially limiting generalizability to other state Medicaid programs and not capturing the full spectrum of policy approaches implemented across all state Medicaid programs. However, these states represent diverse geographic regions and policy approaches while serving approximately 36 million beneficiaries.

The 2018-2023 timeframe encompasses significant policy changes related to COVID-19 public health emergency provisions and Medicaid continuous enrollment requirements, which may have influenced coverage patterns and implementation approaches during the study period. This analysis documents formal policy specifications but does not assess actual implementation experiences, clinician network adequacy, or patient access patterns, which may vary substantially from written policy requirements.

## Conclusions

The Medicaid policy variation documented in this economic evaluation establishes a foundation for future research examining how coverage differences may affect patient access and outcomes. States that invest in comprehensive, integrated coverage models provide opportunities for comparative effectiveness research that other states can use to inform evidence-based policy development. The taxonomic framework developed in this analysis enables systematic evaluation of different policy approaches and their impacts on care delivery for patients with co-occurring chronic pain and OUD.

## Notes

Supplement.
Data Sharing Statement
