# Supplementary material for: Medicaid Coverage Policy Variations for Chronic Pain and Opioid Use Disorder Treatment
Source: JAMA Netw Open. 2025 Aug 13;8(8):e2526796. doi: 10.1001/jamanetworkopen.2025.26796 (PMC12351411; doi:10.1001/jamanetworkopen.2025.26796)
Supplement: Supplement. — Data Sharing Statement [file jamanetwopen-e2526796-s001.pdf]

# Data Sharing Statement

Adams. Medicaid Coverage Policy Variations for Chronic Pain and Opioid Use Disorder Treatment. *JAMA Netw Open*. Published August 13, 2025.
doi:10.1001/jamanetworkopen.2025.26796

**Data**
**Data available:** No

**Additional Information**
**Explanation for why data not available:** Publicly available data at the time of submission.
